# Effect of Vitamin D and Skeletal Muscle Mass on Prognosis of Patients with Diffuse Large B-Cell Lymphoma

**DOI:** 10.3390/nu16162653

**Published:** 2024-08-11

**Authors:** Nobuhiko Nakamura, Nobuhiro Kanemura, Takuro Matsumoto, Hiroshi Nakamura, Yuhei Shibata, Kimihiro Yamaguchi, Junichi Kitagawa, Yoshikazu Ikoma, Tomomi Suzaki, Yuto Kaneda, Soranobu Ninomiya, Eri Takada, Takeshi Hara, Hisashi Tsurumi, Masahito Shimizu

**Affiliations:** 1 Department of Hematology and Infectious Disease, Gifu University Hospital, Gifu 501-1194, Japan; nkane@orion.ocn.ne.jp (N.K.); matsutakeseven@yahoo.co.jp (T.M.); ayapokopoko2@gmail.com (H.N.); ikoma.yoshikazu.g1@f.gifu-u.ac.jp (Y.I.); yuto-hayashi@outlook.com (Y.K.); tsurumi.hisashi.v1@f.gifu-u.ac.jp (H.T.); shimizu.masahito.j1@f.gifu-u.ac.jp (M.S.); 2 Department of Hematology, Gifu Municipal Hospital, Gifu 500-8513, Japan; shiba4174@hotmail.co.jp (Y.S.); chris_kimihiro@yahoo.co.jp (K.Y.); jkitagawa1128@gmail.com (J.K.); tomomi2154@gmail.com (T.S.); 3 Ninomiya Clinic, Gifu 504-0941, Japan; soranobu2000@yahoo.co.jp; 4 Department of Hematology, Seino Kosei Hospital, Gifu 501-0532, Japan; nyentakada@yahoo.co.jp; 5 Department of Hematology, Matsunami General Hospital, Gifu 501-6062, Japan; haratake@muh.biglobe.ne.jp

**Keywords:** diffuse large B-cell lymphoma, vitamin D, sarcopenia, skeletal muscle index, international prognostic index

## Abstract

This study investigated the prognostic impact of vitamin D deficiency and reduced skeletal muscle mass in diffuse large B-cell lymphoma (DLBCL) patients. A retrospective analysis of 186 newly diagnosed DLBCL patients from 2012 to 2022 was conducted, measuring serum 25-hydroxyvitamin D [25(OH)D] levels and the skeletal muscle index (SMI). Decreased vitamin D levels were linked to more severe DLBCL disease, with a median 25(OH)D concentration of 13 (4.0–27) ng/mL. Males in the group with a low SMI had a considerably lower 25(OH)D concentration. The optimal threshold of 25(OH)D levels for overall survival (OS) was 9.6 ng/mL, with lower values associated with a higher likelihood of recurrence and mortality. Multivariable analysis showed hazard ratios for OS of 1.4 [95% CI 0.77–2.5] for a low SMI and 3.2 [95% CI 1.8–5.8] for low 25(OH)D concentration. The combination of a low SMI and low vitamin D concentration resulted in the worst prognosis. Thus, low levels of vitamin D associated with disease progression significantly impact DLBCL prognosis, which can be further stratified by the SMI, providing valuable insights for patient management and potential therapeutic interventions.

## 1. Introduction

Diffuse large B-cell lymphoma (DLBCL) is the predominant form of lymphoma [1]. The heterogeneity of DLBCL leads to varied responses to standard treatments, resulting in a 5-year overall survival (OS) that ranges from 40 to 60% [2]. The prognosis for DLBCL patients has improved with the use of rituximab, a key antibody agent, being added to traditional chemotherapy regimens [3]. In addition, advances in therapeutic options, such as new regimens with polatuzumab vedotin, are beneficial to patients with DLBCL [4]. However, there remains a need to identify prognostic factors that will allow for patient stratification and personalized treatment approaches to improve prognosis and quality of life further [5].

Sarcopenia is a progressive disorder marked by a reduction in both muscular mass and strength that is typically seen in elderly individuals. Sarcopenia is a notable predictor in several forms of cancer [6]. Patients with DLBCL have also been reported to show a worse prognosis when their skeletal muscle index (SMI) is low at diagnosis, especially males [7]. A systematic review and meta-analysis of DLBCL patients found that sarcopenia is associated with poor OS and progression-free survival (PFS), even after adjusting for confounding factors [8]. Therefore, assessment of sarcopenia helps identify DLBCL patients with a poor prognosis, and the development of prevention and treatment may improve the prognosis of these patients. However, the mechanisms underlying the deterioration of disease in DLBCL patients with sarcopenia have not been fully elucidated.

Vitamin D is an essential fat-soluble vitamin responsible for calcium homeostasis and bone health [9]. In addition, vitamin D and associated signals have a variety of roles in immunomodulation and the control of inflammation [10]. Low levels of vitamin D have been reported to be associated with increased risks of certain cancers and adverse outcomes in patients with various malignancies [11,12]. Insufficient levels of vitamin D have also been linked to a poor prognosis for many hematological malignancies, including DLBCL [13,14]. In addition, some reports have suggested that vitamin D supplementation may improve treatment responsiveness in DLBCL patients [15,16]. However, no specific conclusions have been reached regarding the usefulness of vitamin D measurements in stratifying poor prognosis groups of DLBCL patients and their potential supplementation for improving prognosis.

More recently, there has been an increasing interest in the connection between vitamin D and sarcopenia. Sarcopenia risk is increased in those with low vitamin D levels [17], but its supplementation may be helpful in the prevention and treatment of sarcopenia [18]. Therefore, the coexistence of sarcopenia and vitamin D deficiency may lead to worse pathophysiology and prognosis in various diseases [19]. Still, no studies have analyzed these factors together in DLBCL patients. Thus, the present investigation evaluated vitamin D levels and skeletal muscle mass in DLBCL patients and explored how these factors affect prognosis.

## 2. Materials and Methods

### 2.1. Cohort of Patients

This study analyzed retrospective data from patients (18 years of age or older) newly diagnosed with DLBCL and treated at Gifu University Hospital from February 2012 to April 2022. Histological diagnoses were made according to the 2008 World Health Organization criteria and their revision [20]. The standard regimens in this study included rituximab, cyclophosphamide, doxorubicin, vincristine, and prednisone (R-CHOP) or rituximab, cyclophosphamide, tetrahydropyranil-adriamycin, vincristine, and prednisone (R-THP-COP) [21]. Cases with histological transformation from follicular lymphoma, human immunodeficiency virus-associated lymphoma, methotrexate-associated lymphoproliferative disorder, and primary central nervous system lymphoma were excluded. The study complied with the principles stated in the Declaration of Helsinki, and approval was obtained from the ethics committee of the Gifu University Graduate School of Medicine (approval number 2022-221, approval date: 4 January 2023). Because the study was retrospective, the ethics committee waived the need for informed consent.

### 2.2. Demographic and Clinical Information

Study variables included sex, age, Eastern Cooperative Oncology Group Performance Status (ECOG PS), B symptoms, which include fever > 38 °C, night sweats, and unexplained weight loss > 10% of body weight over six months, serum lactate dehydrogenase (LDH) level, Ann Arbor stage, number of extranodal lesions, soluble interleukin two receptor (sIL2-R) level, and the International Prognostic Index (IPI) [22]. Patients were categorized into two age groups: ≤60 years and >60 years. The age cut-off of 60 years was selected based on its established use in the IPI [22]. Complete response (CR) and partial response rates were combined into an overall response (OR) rate. The Hans method was used to determine the cell-of-origin (COO) of DLBCL by immunohistochemistry-based subtyping [23]. Outcome variables included best response, relapse or disease progression after treatment, and death. The response was evaluated according to the Cheson criteria, 2007 [24]. The attending physician determined treatment.

### 2.3. Measurement of Serum 25-Hydroxyvitamin D [25(OH)D] Levels

The electrochemiluminescence method was used to measure the levels of 25-hydroxyvitamin D [25(OH)D] in cryopreserved serum within 40 days of diagnosis [25]. The laboratory reported the lowest value for data below 4.0 ng/mL. For statistical reasons, values below 4.0 ng/mL were considered and scored as 4.0 ng/mL. To conduct the study, 25 (OH)D levels were divided into three categories: deficient (less than 20 ng/mL), insufficient (20–29 ng/mL), and standard (30 ng/mL or over) [26].

### 2.4. Measurement of Body Composition

Skeletal muscle mass was measured using CT images at diagnosis, as in previous studies [7]. The SMI was calculated by dividing the muscle mass of the third lumbar vertebra by the square of the individual’s height (cm^2^/m^2^). The CT scans were examined with SliceOmatic (version 5.0; Tomovision, Montreal, QC, Canada) to identify and isolate specific tissues based on pre-determined Hounsfield unit (HU) ranges. ABACS (Automatic Body Composition Analyzer using Computed Tomography image Segmentation) software was used according to previously established procedures to reduce interobserver measurement error [27]. This software recognizes muscle tissue based on its radiodensity, which falls from −29 to +150 HU (Figure S1). To avoid misidentifying organs as muscle tissue, the software included details about the shape of the L3 muscle, since organs also have a similar radiodensity range [28]. The patients were categorized into groups with low and high SMI using sex-specific cutoffs recommended by the Japanese Society of Hepatology guidelines: 42 cm^2^/m^2^ for males and 38 cm^2^/m^2^ for females [29].

Visceral adipose tissue was defined as tissue lying inside the border of the defined muscle area with a radiodensity between −150 and −50 HU. Subcutaneous adipose tissue was defined as tissue lying outside the border of the defined muscle area with a radiodensity of between −190 and −30 HU. Each value was divided by the square of the height to obtain the visceral adipose tissue index (VATI) and subcutaneous adipose tissue index (SATI) [30].

### 2.5. Statistical Analysis

Data distributions are shown comprehensively by providing continuous variables’ median (range) and mean (standard deviation) values. The Mann–Whitney U test or the Kruskal–Wallis test was used to compare groups for these variables due to their non-normal distributions. Categorical variables are presented using numerical and percentage values. The Chi-squared or Fisher’s exact test was used to compare groups, as appropriate. PFS and OS were the primary endpoints of interest. OS was described as the period from the start of treatment to the last follow-up or death from any cause. PFS was the time from the beginning of treatment to the first occurrence of disease progression, relapse after an initial good response, or death from any cause. Patients who were no longer being monitored at time of the last observation were discontinued. Survival curves were estimated using the Kaplan–Meier method and compared by the log-rank test. Median follow-up was determined based on the reverse Kaplan–Meier method and is presented as median and interquartile range values. The statistical analyses were performed using EZR version 1.61 [31], with a significance level of two-sided *p* < 0.05.

The cutoff values of 25(OH)D for predicting 5-year OS and 5-year PFS were estimated using receiver-operating characteristic (ROC) curves [32]. The ROC curve analysis included patients who survived or were censored for more than five years after treatment and died within five years of diagnosis. Previously recognized traditional predictors of DLBCL were analyzed using multivariable Cox proportional hazards models to determine their association with OS or PFS. The factors considered for the multivariable analysis were sex, COO of DLBCL, IPI, 25(OH)D, and sarcopenia. These predictors were also used as covariates. The differential effect of sarcopenia on survival related to 25(OH)D was examined using a restricted cubic spline (RCS) with 4 knots to assess the non-linear correlation with OS. The number of knots was found using Akaike’s information criterion.

## 3. Results

### 3.1. Patients’ Characteristics

**Figure 1:**
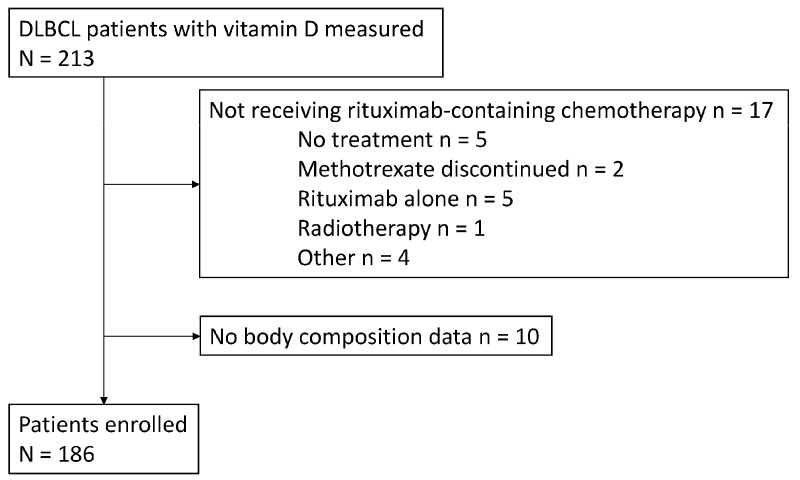
Patient flow. DLBCL: diffuse large B-cell lymphoma.

Of the 213 patients with DLBCL, 186 were included in the analysis after 17 patients who did not receive rituximab-containing chemotherapy and 10 who did not have appropriate CT images were excluded (Figure 1). Table 1 provides a summary of the patients’ characteristics. The patients’ ages ranged from 20 to 93 years, with a median of 71 years. In addition, 80% of the patients were over the age of 60 years. This age distribution is consistent with the known epidemiology of DLBCL, which typically presents at a median age of approximately 70 years [33]. There were 106 (57%) male patients, 27 (15%) with ECOG PS ≥ 2, and 105 (57%) with Ann Arbor staging III-IV. When classified by the IPI category, 56 (30%) were low risk, 41 (22%) were low-intermediate risk, 41 (22%) were high-intermediate risk, and 48 (26%) were high risk. For initial treatment, 158 (85%) patients underwent R-CHOP.

### 3.2. Comparison of SMI and Vitamin D Levels

The median SMI was 40 cm^2^/m^2^ in males (7–76 cm^2^/m^2^) and 34 cm^2^/m^2^ (7–63 cm^2^/m^2^) in females. Of the males, 38 (36%) had a low SMI, whereas, of the females, 45 (56%) had a low SMI. The median vitamin D level of all patients was 13 ng/mL (4.0–27 ng/mL); 35 (19%) were deficient in vitamin D, 151 (81%) had insufficient vitamin D levels, and none had normal vitamin D levels.

Table 2 shows 25(OH)D levels categorized by patients’ features. Differences in 25(OH)D were observed for all IPI items, with significantly lower levels with ECOG PS ≥ 2, Ann Arbor Stage III/IV, elevated LDH, and high IPI, but significantly higher levels with age > 60 years. In addition, patients with B symptoms had significantly lower vitamin D levels. Eleven (14%) female patients took active vitamin D formulations. However, there was no discernible variation in vitamin D levels between patients taking and not taking the preparations.

**Figure 2:**
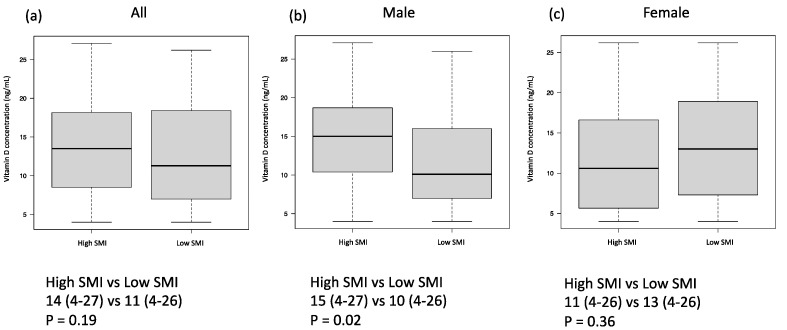
Comparison of vitamin D levels in patients with a high SMI and low SMI. All cases (**a**), male (**b**), and female (**c**). SMI: skeletal muscle index.

Between those with low and high SMIs, there was no significant difference in 25(OH)D levels (*p* = 0.19, Figure 2a). Nevertheless, males with lower SMIs had significantly lower vitamin D levels (*p* = 0.02, Figure 2b), whereas no significant difference was seen in females (*p* = 0.36, Figure 2c). Examining the correlations between 25(OH)D and body mass index (BMI), SMI, VATI, and STAI by sex showed a positive correlation (r = 0.21, *p* = 0.03) between 25(OH)D and SMI in males, but a negative correlation (r = −0.23, *p* = 0.04) was seen in females. Additionally, 25(OH)D levels were not correlated with BMI, VATI, or SATI in either sex (Figure S2).

### 3.3. Patient Survival Analysis by Skeletal Muscle Mass and Vitamin D Levels

**Figure 3:**
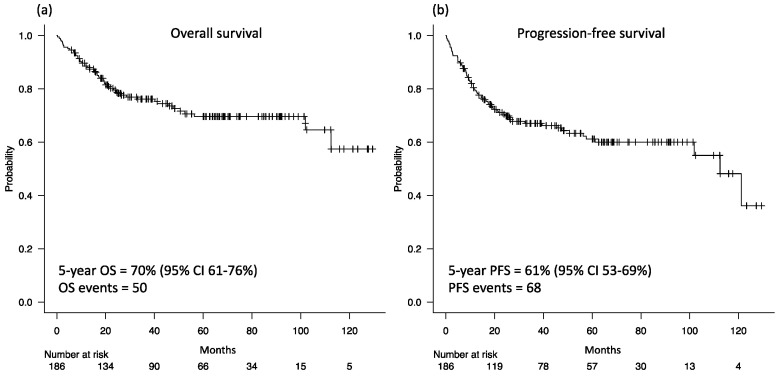
Kaplan–Meier curves for overall survival (**a**) and progression-free survival (**b**) of all patients. OS: overall survival; PFS: progression-free survival; CI: confidence interval.

**Figure 4:**
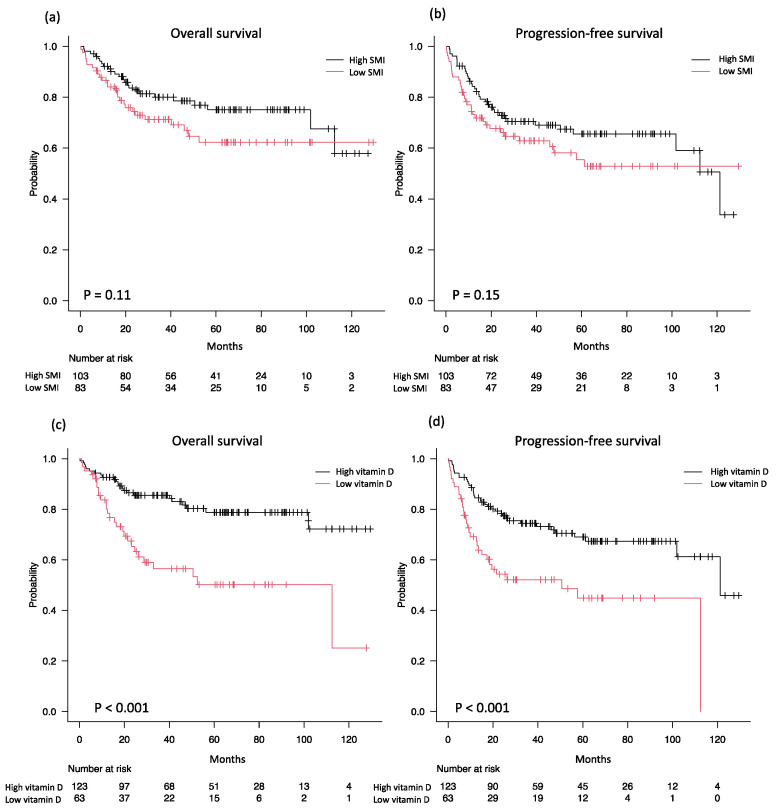
Kaplan–Meier curves by SMI of overall survival (**a**) and progression-free survival (**b**). Overall survival (**c**) and progression-free survival (**d**) by vitamin D level. SMI: skeletal muscle index.

**Figure 5:**
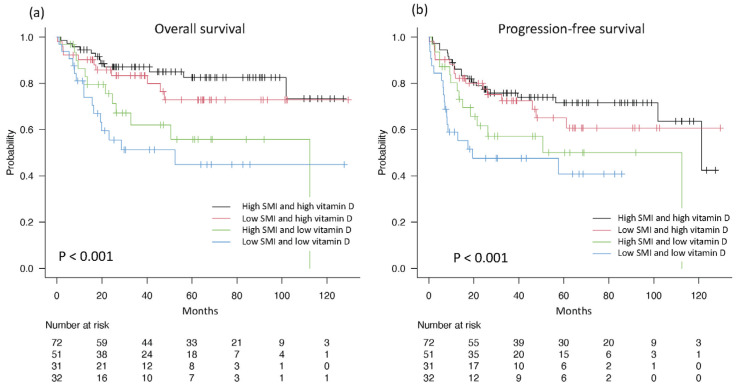
Kaplan–Meier curves for SMI and vitamin D combined for overall survival (**a**) and progression-free survival (**b**). SMI: skeletal muscle index.

The median overall observation period was 60 months (interquartile range 29–83 months), with a 5-year OS of 70% [95% confidence interval (CI) 61–76%, Figure 3a] and 5-year PFS of 62% (95% CI 53–69%, Figure 3b). The low-SMI group had lower 5-year OS (62% vs. 75%, *p* = 0.11, Figure 4a) and 5-year PFS (56% vs. 66%, *p* = 0.15, Figure 4b) than the high-SMI group, but these differences were not significant.

In the ROC curve analyses, the ideal cutoff value of 25(OH)D for predicting OS and PFS was 9.6 ng/mL (Figure S3); 63 (34%) patients were in the low-vitamin-D group [25(OH)D ≤ 9.6 ng/mL] and 123 (66%) were in the high-vitamin-D group [25(OH)D > 9.6 ng/mL]. The low-vitamin-D group had a substantially higher prevalence of cases with ECOG PS > 2, Ann Arbor stage III/IV, increased LDH, and extranodal sites ≥ 2 compared with the high-vitamin-D group (Table 3). The low-vitamin-D group also tended to have more B symptoms and a higher proportion of high-intermediate or high-risk IPIs, but these differences were not significant. The low-vitamin-D group had significantly lower 5-year OS (50% vs. 79%, *p* < 0.001, Figure 4c) and 5-year PFS (45% vs. 69%, *p* < 0.001, Figure 4d) than the high-vitamin-D group. The group with low vitamin D levels tended to have a lower CR rate and a considerably lower OR rate. Furthermore, the low-vitamin-D group also had a significantly higher recurrence rate and a substantially higher number of deaths than the high-vitamin-D group. Nevertheless, the two groups showed no significant difference in cause of death (Table 4).

When patients were categorized into four groups based on their SMI and vitamin D levels, the prognosis was the worst for patients with a low SMI and low vitamin D levels. These patients had a 5-year OS rate of 44% and a 5-year PFS rate of 41%. In contrast, individuals with high SMI and high vitamin D levels showed the best prognosis, with a 5-year OS rate of 83% and a 5-year PFS rate of 72%. The observed differences were significant with a high level of confidence (*p* < 0.001), as demonstrated in Figure 5.

### 3.4. Multivariable Analysis of Clinical Factors Associated with Patient OS and PFS

A multivariable analysis using the COX proportional hazards model examined the relationships between several factors (sex, COO, IPI, low vitamin D level, and low SMI) and survival. It showed that those with low vitamin D levels had an HR of 3.2 (95% CI 1.8–5.8, *p* < 0.001) for OS. Similarly, for PFS, the HR was 2.5 (95% CI 1.5–4.1, *p* < 0.001). In contrast, the HR for OS with a low SMI was 1.4 (95% CI 0.77–2.5, *p* = 0.28), and, for PFS, it was 1.4 (95% CI 0.86–2.4, *p* = 0.17; Table 5).

### 3.5. Effects of Skeletal Muscle Mass and Vitamin D Levels on OS and PFS on Restricted Cubic Spline Analysis

**Figure 6:**
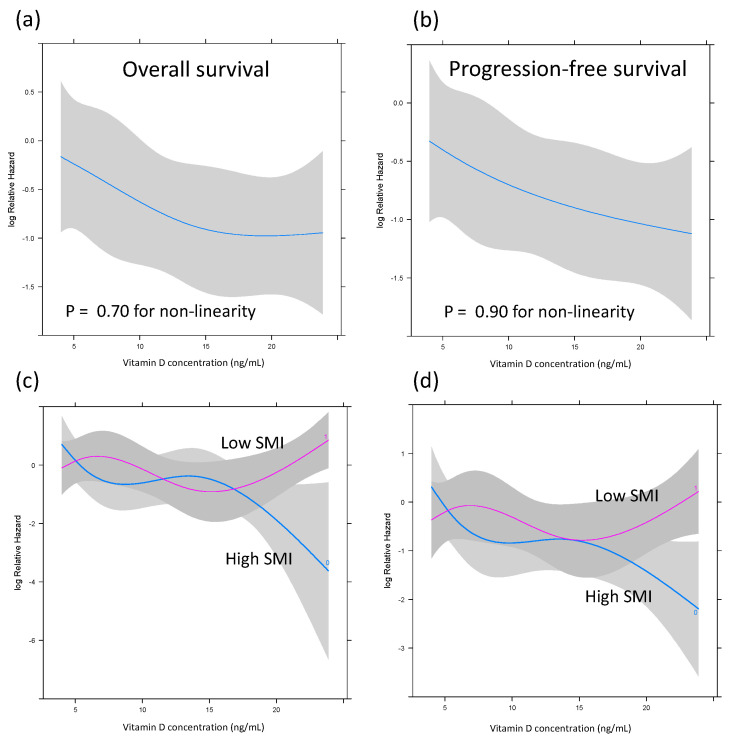
Relationships between vitamin D and overall survival (**a**) and progression-free survival (**b**) with the Cox proportional hazards model using restricted cubic splines. Comparison of the impact of vitamin D on overall survival (**c**) and progression-free survival (**d**) across two groups stratified by SMIs using a covariate-adjusted restricted cubic spline hazard model. The solid line depicts the log hazard ratio and the shaded area represents the 95% confidence interval. OS: overall survival; PFS: progression-free survival; SMI: skeletal muscle index.

The RCS analysis showed that the HR for OS decreased until the 25(OH)D level reached 15 ng/mL and remained constant (Figure 6a). In contrast, there was a clear and direct relationship between the vitamin D level and PFS. The HR for PFS decreased constantly as the vitamin D level increased, as shown in Figure 6b. When the effect of SMI status was added to the examination, the HRs for OS and PFS did not decrease with the increasing vitamin D in the low-SMI group. However, the HRs for OS and PFS decreased with increasing vitamin D in the high-SMI group (Figure 6b,c).

### 3.6. Association of BMI and VATI with Survival

A post hoc analysis to examine the associations of BMI and VATI with survival was conducted using the Kaplan–Meier method. The BMI was classified based on the following standards established by the WHO: individuals with a BMI less than 18.5 kg/m^2^ were considered underweight, those with a BMI between 18.5 and 24.9 kg/m^2^ were considered to have an average weight, those with a BMI between 25 and 29.9 kg/m^2^ were classified as overweight, and those with a BMI equal to or more than 30 kg/m^2^ were considered obese. No significant differences were observed in OS (*p* = 0.60) or PFS (*p* = 0.41) among these BMI groups (Figure S4a,b). Patients were classified into high and low VATI groups based on the median VATI value of 31 cm^2^/m^2^. No significant differences were found in OS (*p* = 0.86) or PFS (*p* = 0.11) between these groups (Figure S4c,d).

## 4. Discussion

This study is the first to examine the influence of skeletal muscle mass and low vitamin D levels on the prognoses of patients with DLBCL. The present study demonstrated that most patients with DLBCL are vitamin D-deficient. None of the patients in the present study group had normal vitamin D levels, consistent with the standard Japanese population observation of vitamin D deficiency. According to recent research, 98% of adult Japanese people do not have enough vitamin D [34]. Nevertheless, it is impossible to exclude the possibility that DLBCL affects vitamin D status. Decreased vitamin D levels were also strongly associated with a worse DLBCL stage. Low-vitamin-D patients had a significantly higher recurrence rate and worse prognoses. These findings align with other studies that have shown a correlation between low levels of vitamin D and a poor prognosis in DLBCL [15,35,36]. The vitamin D threshold in the present study was set at 9.6 ng/mL, but reports of this threshold have varied (range: 9–40 ng/mL) [13,14]. Because blood levels of vitamin D have been reported to be significantly affected by region of residence, race, and lifestyle [37], these factors may need to be considered when establishing an appropriate threshold.

The prognosis of patients with DLBCL is affected by sarcopenia and skeletal muscle depletion [8]. This cohort also showed a trend toward worse survival for patients with lower SMI, but the difference was not significant. Similarly, the post hoc analysis found no significant associations between BMI or VATI and survival outcomes in DLBCL patients, suggesting that the effect of body composition on the prognosis of DLBCL patients may be complex and multifaceted. However, in the RCS analysis, a decrease in HR with increasing vitamin D levels was observed in patients with a high SMI, whereas no such decrease was observed in patients with a low SMI. These findings may indicate that the biological activity of vitamin D is lower in DLBCL patients with low muscle mass, making it challenging to obtain the benefit of vitamin D, i.e., improved prognosis. Because muscle tissue serves as a dynamic storehouse of vitamin D [38,39], patients with low muscle mass may have reduced vitamin D storage and subsequently lower bioavailability for various physiological processes such as immunomodulation and inflammation [40]. The present study also demonstrated that persons with both low vitamin D levels and low SMIs have the worst prognoses, suggesting the existence of a synergistic interaction between these pathologies that worsens the prognosis. These findings emphasize the need for comprehensive patient assessment that considers nutritional status and skeletal muscle mass when planning treatment for DLBCL patients.

The present study found a sex-specific correlation between the vitamin D level and the SMI. In males, there was a positive correlation between the vitamin D level and the SMI, suggesting that higher vitamin D levels may be protective against skeletal muscle depletion. This is consistent with previous studies showing that vitamin D supplementation can improve muscle strength and function in elderly persons [41,42]. However, female SMI and vitamin D levels were negatively correlated, and this result was the same in analyses excluding females taking oral active vitamin D preparations. This discrepancy may result from differences in vitamin D production and metabolism between males and females. Studies evaluating plasma 25(OH)D levels and the effects of vitamin D on diseases have shown significant differences between males and females [43]. An observational study found a direct relationship between vitamin D levels and the amount of muscle mass in men diagnosed with sarcopenia [44]. A separate investigation including 65-year-olds showed a relationship between low vitamin D levels, walking speed, and minimum hand grip strength that was more significant in the male group than in the female group [45]. Additional investigation is required to clarify the complex correlation between vitamin D and muscle health, which could improve our understanding of the prevention and treatment of skeletal muscle loss in both sexes.

The importance of skeletal muscle mass in cancer prognosis extends beyond DLBCL. A growing body of evidence suggests that low skeletal muscle mass is associated with poor outcomes across various cancer types [46]. For example, in breast cancer, a correlation has been shown between a low skeletal muscle mass and a reduced survival rate [47]. Similarly, in lung cancer, sarcopenia is associated with increased treatment-related toxicities and decreased survival [48]. The mechanisms underlying these associations are multifaceted, including altered drug pharmacokinetics, increased systemic inflammation, and reduced functional capacity [49]. The interaction between skeletal muscle mass and treatment outcomes observed in the present DLBCL cohort aligns with findings in other cancer types. Importantly, interventions aimed at improving physical function through exercise have shown promise in various cancer populations [50]. This underscores the potential importance of considering skeletal muscle mass and physical activity in comprehensive cancer care strategies. However, it is crucial to acknowledge that the specific impact of skeletal muscle and vitamin D may vary across cancer types due to differences in disease biology, treatment modalities, and patient populations. The present findings in DLBCL patients contribute to this broader understanding while highlighting the need for cancer-specific investigations.

The present study’s findings have implications for how vitamin D supplementation and maintaining skeletal muscle may help persons with DLBCL, particularly those with sarcopenia, by improving their pathophysiology. In DLBCL patients who underwent R-CHOP therapy, patients with normalized 25(OH)D levels after vitamin D supplementation have been reported to show better event-free survival than those with persistent deficiencies [16]. Vitamin D deficiency impairs the cytotoxic activity of rituximab in lymphoma cells, which is facilitated by vitamin D supplementation [15]. Therefore, maintaining adequate vitamin D levels may enhance the efficacy of rituximab in patients with DLBCL. Based on these findings, a multifaceted approach involving vitamin D supplementation and interventions to increase muscle mass may benefit DLBCL patients, particularly those with sarcopenia. Evidence from other groups showed that resistance training combined with sufficient protein intake can successfully improve muscle mass and strength in sarcopenic individuals [51]. However, the efficacy and safety of such interventions in DLBCL patients, particularly during active treatment, require careful investigation. Future studies should explore the synergistic effects of vitamin D supplementation along with tailored exercise and nutritional interventions for DLBCL patients with sarcopenia, considering their overall condition and treatment plan. However, the appropriate concentration of vitamin D for clinical effectiveness, the methods of its administration, and whether vitamin D supplementation improves the long-term prognosis of DLBCL patients are unknown and require further detailed investigation. In addition, both nutritional and exercise therapies are basic countermeasures against sarcopenia, but their efficacy in DLBCL has not been fully reported. Consequently, more research is required to assess the therapeutic benefit of these treatments in DLBCL patients, particularly if they enhance prognosis and reduce morbidity.

The present study has several limitations. First, this was a single-center, retrospective study, and the possibility of bias affecting the relationships of vitamin D and SMI with prognosis cannot be excluded. Second, other factors affecting muscle health, such as physical activity levels, dietary intake, and other nutritional deficiencies, were not evaluated. Third, sarcopenia could not be strictly assessed because muscle strength was not measured. However, despite these limitations, the present study has some advantages, including assessment of body mass composition, risk analysis using RCS, and multivariate models that strengthen the evidence.

## 5. Conclusions

In conclusion, the results suggest a potential synergistic effect between vitamin D deficiency and skeletal muscle depletion on the outcomes of DLBCL patients. Low vitamin D levels are more predictive for DLBCL patients than the SMI, and the SMI may be used to stratify the patients further. These findings emphasize the significance of evaluating these patients’ nutritional and muscular health. Prospective studies are necessary to confirm the current findings and investigate the potential benefits of vitamin D therapy and its administration for the prevention or treatment of sarcopenia in DLBCL patients.

## Figures and Tables

**Table 1:** Patients’ characteristics at diagnosis (descriptive statistics).

	All Patients (*n* = 186)
Age, y—median (range); mean (SD)	71 (20–93); 69 (13)
Age > 60 y—*n* (%)	148 (80)
Male—*n* (%)	106 (57)
ECOG PS ≥ 2—*n* (%)	27 (15)
B symptoms—*n* (%)	49 (26)
BMI (kg/cm^2^)—median (range); mean (SD)	22 (15–41); 22 (4)
SMI (cm^2^/cm^2^)—median (range); mean (SD)	42 (24–76); 42 (8)
VATI (cm^2^/cm^2^)—median (range); mean (SD)	31 (2–120); 35 (26)
SATI (cm^2^/cm^2^)—median (range); mean (SD)	37 (1–140); 42 (25)
Low SMI—*n* (%)	83 (45)
sIL-2R (U/mL)—median (range); mean (SD)	1200 (190–91000); 3000 (7300)
Vitamin D (ng/mL)—median (range); mean (SD)	13 (4.0–27); 13 (7)
Extranodal sites ≥ 2—*n* (%)	55 (30)
Ann Arbor Stage III/IV—*n* (%)	105 (57)
Elevated LDH (>ULN)—*n* (%)	116 (62)
COO—*n* (%)	
GCB	69 (37)
Non-GCB	85 (46)
Unknown	32 (17)
IPI—*n* (%)	
Low risk (0, 1)	56 (30)
Low intermediate risk (2)	41 (22)
High intermediate risk (3)	41 (22)
High risk (4, 5)	48 (26)
First treatment—*n* (%)	
R-CHOP	158 (85)
R-THP-COP	25 (13)
R-CVP	3 (2)
Intrathecal therapy—*n* (%)	37 (20)
Radiation therapy—*n* (%)	49 (26)

BMI, body mass index; COO, cell-of-origin; ECOG PS, Eastern Cooperative Oncology Group Performance Status; GCB, germinal center B-cell-like; IPI, International Prognostic Index; LDH, lactate dehydrogenase; R-CHOP, rituximab, cyclophosphamide, doxorubicin, vincristine, prednisone; R-CVP, rituximab, cyclophosphamide, vincristine, prednisone; R-THP-COP, rituximab, cyclophosphamide, tetrahydropyranil-adriamycin, vincristine, prednisone; SATI, subcutaneous adipose tissue index; SD, standard deviation; SMI, skeletal muscle index; sIL-2R, soluble interleukin two receptors; VATI, visceral adipose tissue index.

**Table 2:** Comparison of vitamin D levels by background factors (Mann–Whitney U test or Kruskal–Wallis test).

		Vitamin D (ng/mL)		
	No.	Median (Range)	Mean (SD)	*p*-Value
Age, y				
≤60	38	9.3 (4.0–24)	11 (6)	0.008
>60	148	14 (4.0–27)	14 (7)	
Sex				
Male	106	13 (4.0–27)	14 (6)	0.22
Female	80	12 (4.0–26)	13 (7)	
ECOG PS				
0–1	159	13 (4.0–27)	14 (6)	0.005
≥2	27	7.5 (4.0–26)	10 (6)	
B symptoms				
Absent	137	14 (4.0–27)	14 (6)	0.004
Present	49	10 (4.0–26)	11 (7)	
SMI status				
High SMI	103	14 (4.0–27)	14 (6)	0.19
Low SMI	83	11 (4.0–26)	13 (7)	
Extranodal sites				
0–1	131	14 (4.0–26)	14 (6)	0.001
≥2	55	10 (4.0–27)	11 (6)	
Ann Arbor Stage				
I/II	81	16 (4.0–26)	15 (6)	0.001
III/IV	105	11 (4.0–27)	12 (6)	
LDH				
Normal	70	15 (4.0–26)	15 (6)	0.004
Elevated	116	12 (4.0–27)	12 (7)	
COO				
GCB	69	13 (4.0–26)	14 (7)	0.34
Non-GCB	85	12 (4.0–27)	13 (7)	
Unknown	32	8 (4.0–24)	12 (6)	
IPI				
Low (0, 1)	56	16 (4.0–26)	15 (6)	0.004
Low intermediate (2)	41	14 (4.0–26)	14 (7)	
High intermediate (3)	41	11 (4.0–24)	12 (6)	
High (4, 5)	48	11 (4.0–27)	12 (6)	

COO, cell-of-origin; ECOG PS, Eastern Cooperative Oncology Group Performance Status; GCB, germinal center B-cell-like; IPI, International Prognostic Index; LDH, lactate dehydrogenase; SD, standard deviation; SMI, skeletal muscle index. The Mann–Whitney U test was used to compare two groups (e.g., sex, ECOG PS, B symptoms). The Kruskal–Wallis test was used to compare three or more groups (e.g., COO, IPI categories).

**Table 3:** Patients’ characteristics by vitamin D status (Mann–Whitney U test, Fisher’s exact test, or Chi-squared test).

	High Vitamin D (>9.6 ng/dL) (*n* = 123)	Low Vitamin D (≤9.6 ng/dL) (*n* = 63)	*p*-Value
Age, y—median (range); mean (SD)	72 (21–93); 70 (11)	68 (20–86); 66 (14)	0.12
Age > 60 y—*n* (%)	105 (85)	43 (68)	0.01
Male—*n* (%)	73 (59)	33 (52)	0.43
ECOG PS ≥ 2—*n* (%)	13 (11)	14 (22)	0.05
B symptoms—*n* (%)	27 (22)	22 (35)	0.08
BMI (kg/cm^2^)—median (range); mean (SD)	22 (15–41); 22 (4)	22 (15–33); 23 (4)	0.33
SMI (cm^2^/cm^2^)—median (range); mean (SD)	42 (24–76); 42 (8)	40 (28–63); 42 (8)	0.70
VATI (cm^2^/cm^2^)—median (range); mean (SD)	31 (2–120); 39 (24)	32 (3–120); 46 (27)	0.82
SATI (cm^2^/cm^2^)—median (range); mean (SD)	36 (3–130); 35 (26)	39 (1–140); 36 (27)	0.08
Low SMI—*n* (%)	51 (42)	32 (51)	0.28
sIL-2R (U/mL)—median (range); mean (SD)	2100 (240–18,000); 2400 (8400)	870 (190–91,000); 4000 (4300)	<0.001
Extranodal sites ≥ 2—*n* (%)	29 (24)	26 (41)	0.02
Ann Arbor Stage III/IV—*n* (%)	63 (51)	42 (67)	0.06
Elevated LDH (>ULN)—*n* (%)	68 (55)	48 (76)	0.01
COO—*n* (%)			
GCB	49 (40)	20 (32)	0.048
Non-GCB	59 (48)	26 (41)	
Unknown	15 (12)	17 (27)	
IPI—*n* (%)			0.07
Low (0, 1)	44 (36)	12 (19)	
Low intermediate (2)	28 (23)	13 (21)	
High intermediate (3)	23 (19)	18 (29)	
High (4, 5)	28 (23)	20 (32)	
First treatment—*n* (%)			0.78
R-CHOP	106 (86)	52 (83)	
R-THP-COP	15 (12)	10 (16)	
R-CVP	2 (2)	1 (2)	
Intrathecal therapy—*n* (%)	24 (20)	13 (21)	0.85
Radiation therapy—*n* (%)	39 (32)	10 (16)	0.02

BMI, body mass index; COO, cell-of-origin; ECOG PS, Eastern Cooperative Oncology Group Performance Status; GCB, germinal center B-cell-like; IPI, International Prognostic Index; LDH, lactate dehydrogenase; R-CHOP, rituximab, cyclophosphamide, doxorubicin, vincristine, prednisone; R-CVP, rituximab, cyclophosphamide, vincristine, prednisone; R-THP-COP, rituximab, cyclophosphamide, tetrahydropyranil-adriamycin, vincristine, prednisone; SATI, subcutaneous adipose tissue index; SD, standard deviation; SMI, skeletal muscle index; sIL-2R, soluble interleukin two receptors; VATI, visceral adipose tissue index. The Chi-squared test was used to examine categorical variables, the Fisher’s exact test was used for categorical variables with expected frequencies less than 5, and the Mann–Whitney U test was used for continuous variables.

**Table 4:** Vitamin D levels and response, relapse, and death (Chi-squared test or Fisher’s exact test).

	All Patients (*n* = 186)	High Vitamin D (>9.6 ng/dL) (*n* = 123)	Low Vitamin D (≤9.6 ng/dL) (*n* = 63)	*p*-Value
CR—*n* (%)	150 (83)	105 (87)	45 (75)	0.06
OR—*n* (%)	166 (92)	115 (95)	51 (85)	0.04
Relapse—*n* (%)	45 (24)	24 (20)	21 (33)	0.047
Death—*n* (%)	49 (26)	22 (18)	27 (43)	<0.001
Death reason—*n* (%)				
Lymphoma	29 (59)	11 (50)	18 (67)	0.26
Other	20 (41)	11 (50)	9 (33)	

CR, complete response; OR, overall response. The Chi-squared test was used to compare groups. A Fisher’s exact test was used when expected frequencies were less than five.

**Table 5:** Multivariable COX proportional hazards analysis for overall survival and progression-free survival.

	Overall Survival		Progression-Free Survival	
Factor	HR (95% CI)	*p*-Value	HR (95% CI)	*p*-Value
Male	1.8 (1.0–3.4)	0.051	2.0 (1.2–3.4)	0.01
COO				
GCB	(reference group)		(reference group)	
Non-GCB	2.1 (1.1–4.0)	0.02	1.7 (1.0–2.8)	0.06
Unknown	0.9 (0.4–2.3)	0.90	0.66 (0.29–1.5)	0.31
IPI, category				
Low risk	(reference group)		(reference group)	
Low-intermediate risk	1.9 (0.7–5.6)	0.24	2.2 (0.92–5.2)	0.08
High-intermediate risk	4.4 (1.7–12)	0.003	4.0 (1.8–8.9)	<0.001
High risk	5.7 (2.3–15)	<0.001	5.3 (2.5–11)	<0.001
Low SMI	1.4 (0.8–2.5)	0.28	1.4 (0.86–2.4)	0.17
Low vitamin D	3.2 (1.8–5.8)	<0.001	2.5 (1.5–4.1)	<0.001

CI, confidence interval; COO, cell-of-origin; GCB, germinal center B-cell-like; IPI, International Prognostic Index; SMI, skeletal muscle index.

## Data Availability

The data supporting the study’s conclusions can be acquired from the corresponding author upon reasonable request.

## Supplementary Materials

The following supporting information can be downloaded at: https://www.mdpi.com/article/10.3390/nu16162653/s1, Figure S1: CT images of the third lumbar vertebrae where body composition was measured. Red indicates muscle, yellow indicates visceral fat, and blue indicates subcutaneous fat. (a) A 78-year-old male with an SMI greater than 42 cm^2^/m^2^ is considered to have a high SMI. (b) A 70-year-old female with an SMI of less than 38 cm^2^/m^2^ is assumed to have a low SMI. SATI: subcutaneous adipose tissue index; SMI: skeletal muscle index; VATI: visceral adipose tissue index. Figure S2: Correlations between 25(OH)D and body composition for all patients, male and female. BMI: body mass index; SATI: subcutaneous adipose tissue index; SMI: skeletal muscle index; VATI: visceral adipose tissue index. Figure S3: Receiver-operating characteristic curve predicting overall survival (a) and progression-free survival (b). AUC: area under the curve. Figure S4: Kaplan–Meier curves according to BMI of overall survival (a) and progression-free survival (b). Overall survival (c) and progression-free survival (d) according to the VATI. BMI: body mass index; VATI: visceral adipose tissue index.
