# Supplementary figures and images for: Effect of Vitamin D and Skeletal Muscle Mass on Prognosis of Patients with Diffuse Large B-Cell Lymphoma
Source: Nutrients. 2024 Aug 11;16(16):2653. doi: 10.3390/nu16162653 (PMC11357194; doi:10.3390/nu16162653)

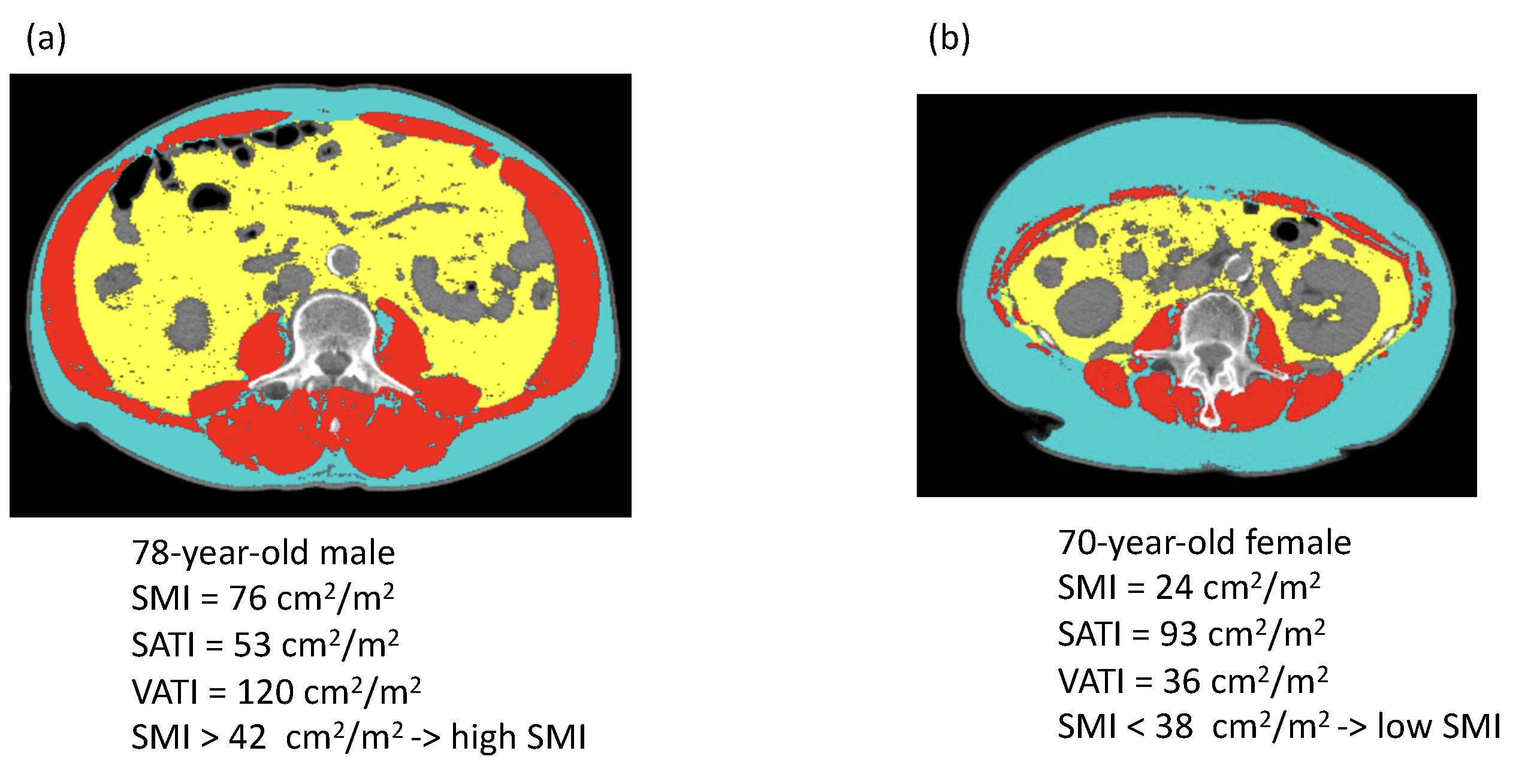

Supplement: Supplementary file 1 [file nutrients-16-02653-s001.zip › Figure S1.tiff]

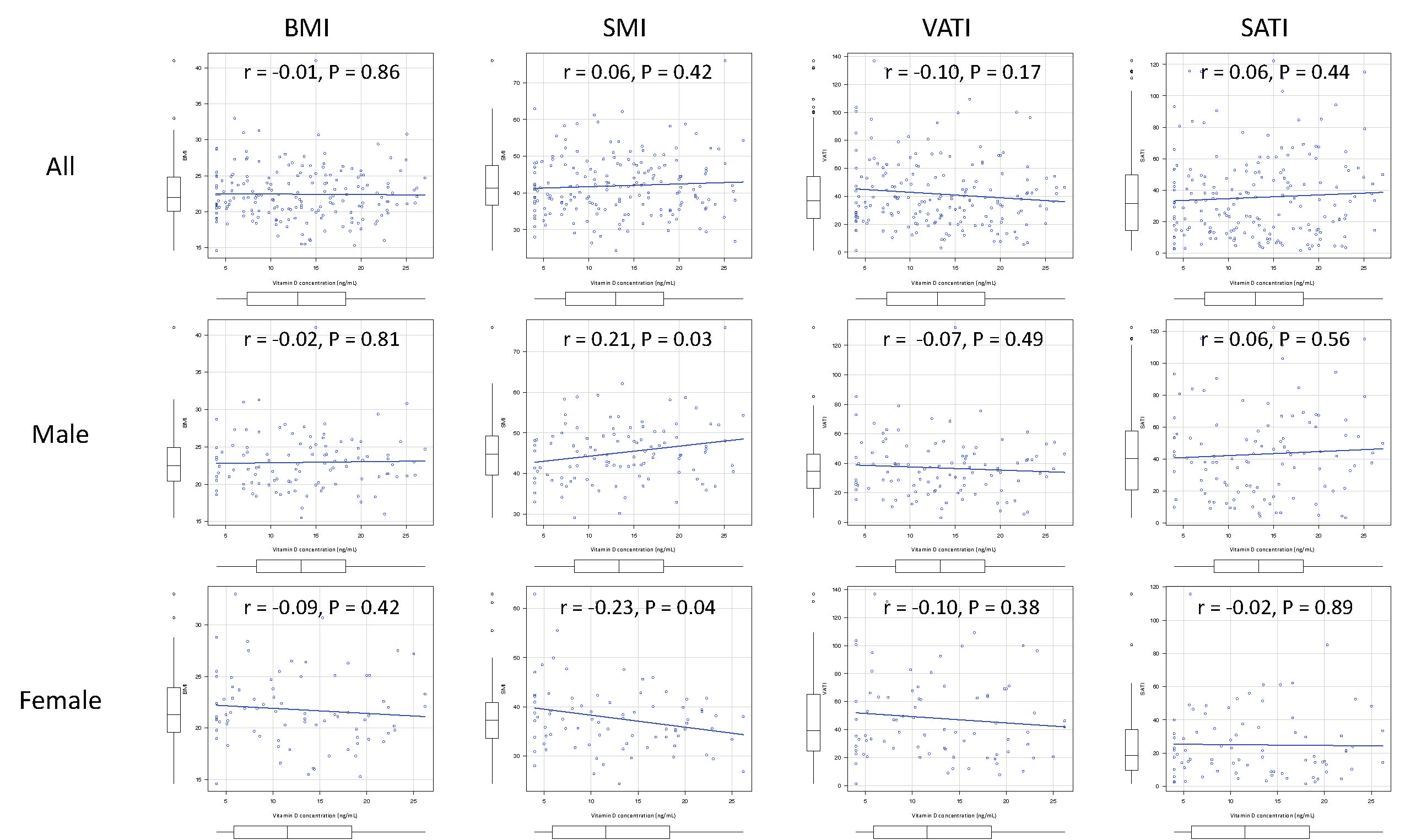

Supplement: Supplementary file 1 [file nutrients-16-02653-s001.zip › Figure S2.tiff]

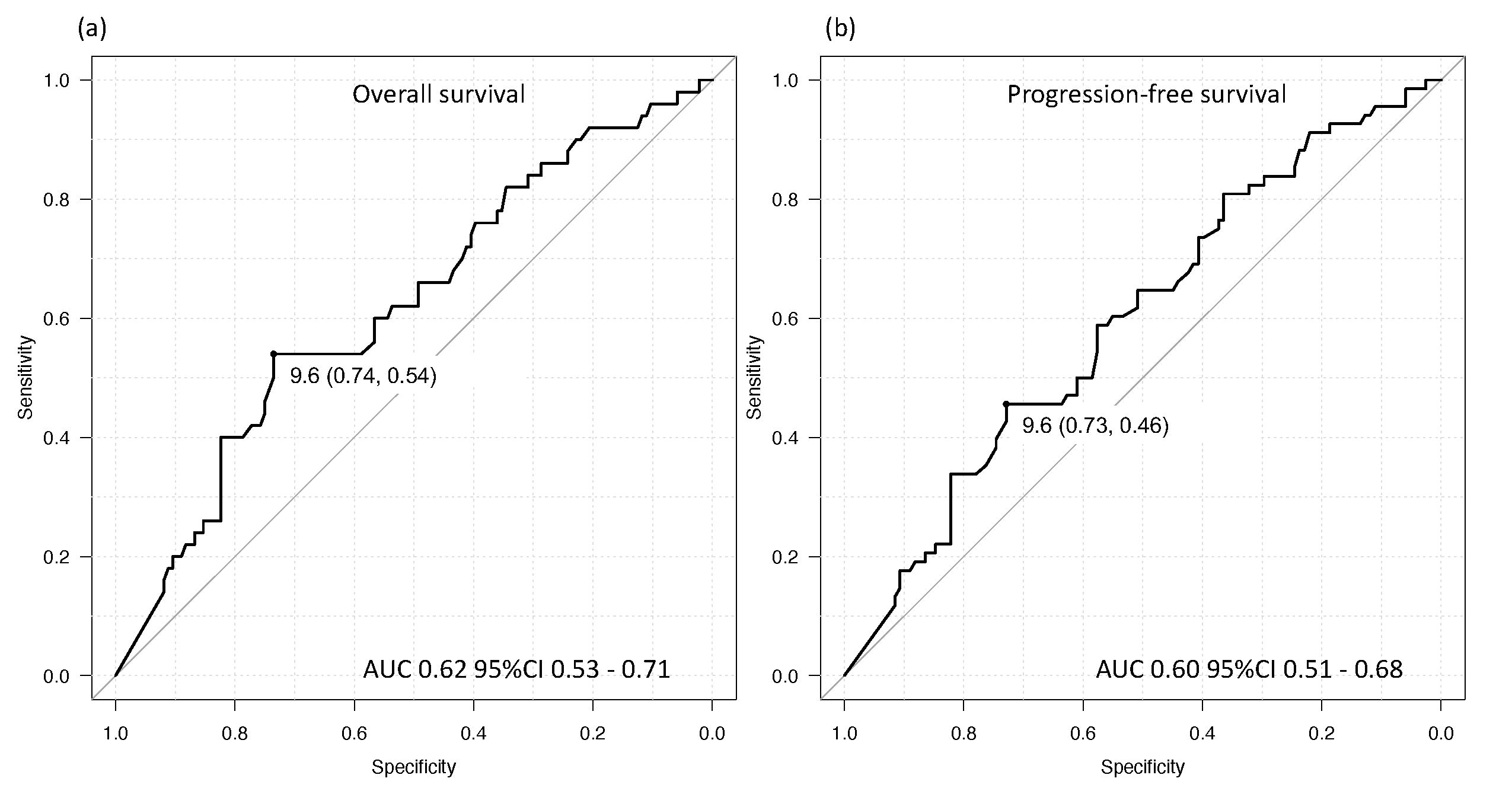

Supplement: Supplementary file 1 [file nutrients-16-02653-s001.zip › Figure S3.tiff]

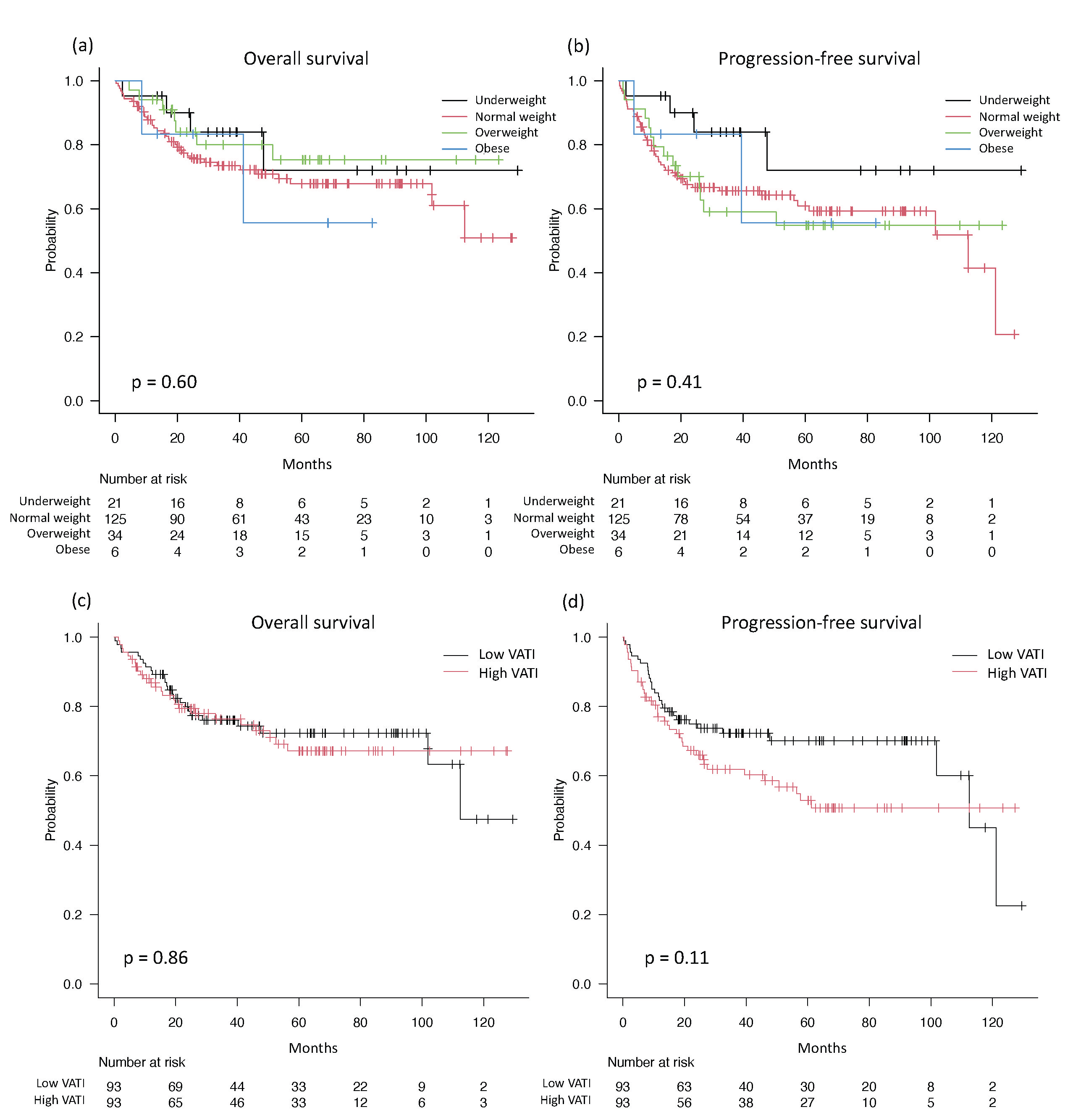

Supplement: Supplementary file 1 [file nutrients-16-02653-s001.zip › Figure S4.tiff]
